# Day-to-day variability in sleep parameters and depression risk: a prospective cohort study of training physicians

**DOI:** 10.1038/s41746-021-00400-z

**Published:** 2021-02-18

**Authors:** Yu Fang, Daniel B. Forger, Elena Frank, Srijan Sen, Cathy Goldstein

**Affiliations:** 1 Michigan Neuroscience Institute, University of Michigan, Ann Arbor, MI USA; 2 Department of Mathematics, University of Michigan, Ann Arbor, MI USA; 3 Department of Computational Medicine and Bioinformatics, University of Michigan, Ann Arbor, MI USA; 4 Michigan Institute for Data Science, University of Michigan, Ann Arbor, MI USA; 5 Department of Psychiatry, University of Michigan, Ann Arbor, MI USA; 6 Department of Neurology, University of Michigan, Ann Arbor, MI USA

**Keywords:** Medical research, Health occupations

## Abstract

While 24-h total sleep time (TST) is established as a critical driver of major depression, the relationships between sleep timing and regularity and mental health remain poorly characterized because most studies have relied on either self-report assessments or traditional objective sleep measurements restricted to cross-sectional time frames and small cohorts. To address this gap, we assessed sleep with a wearable device, daily mood with a smartphone application and depression through the 9-item Patient Health Questionnaire (PHQ-9) over the demanding first year of physician training (internship). In 2115 interns, reduced TST (*b* = −0.11, *p* < 0.001), later bedtime (*b* = 0.068, *p* = 0.015), along with increased variability in TST (*b* = 0.4, *p* = 0.0012) and in wake time (*b* = 0.081, *p* = 0.005) were associated with more depressive symptoms. Overall, the aggregated impact of sleep variability parameters and of mean sleep parameters on PHQ-9 were similar in magnitude (both *r*^2^ = 0.01). Within individuals, increased TST (*b* = 0.06, *p* < 0.001), later wake time (*b* = 0.09, *p* < 0.001), earlier bedtime (*b* = − 0.07, *p* < 0.001), as well as lower day-to-day shifts in TST (*b* = −0.011, *p* < 0.001) and in wake time (*b* = −0.004, *p* < 0.001) were associated with improved next-day mood. Variability in sleep parameters substantially impacted mood and depression, similar in magnitude to the mean levels of sleep parameters. Interventions that target sleep consistency, along with sleep duration, hold promise to improve mental health.

## Introduction

Sleep health is a multidimensional construct that includes parameters beyond sleep duration, such as timing and regularity^1^. Although sleep plays a critical role in general and mental health^2^, studies that have evaluated the role of sleep in various conditions often reduce sleep health to a single, summary parameter (e.g., sleep duration over the course of the night) obtained by a retrospective, subjective query.

However, emerging evidence has identified that stability of the sleep-wake schedule over time is a particularly important contributor to health^3,4^. The predictive value of sleep variability has exceeded that of mean levels of sleep parameters in a variety of medical conditions^3,5,6^. Although disruption to our internal time-keeping system, or circadian rhythm, was recently associated with poor mental health in a study of more than 90,000 individuals^7^, the role of sleep variability, which encompasses behavioral, homeostatic, and circadian contributions to sleep, remains unclear. Previous investigations that evaluated the relationship between increased sleep variability and mental health, whether operationalized as day-to-day shifts over time (intraindividual variability) or weekday-weekend sleep discrepancies (social jet lag), have been limited by self-report measures^3,8,9^ or, if objective measures are used, brief recording duration^10–14^ or small cohort size^10–17^. Therefore, our understanding of the contribution of sleep variability to mental health remains incomplete and minimal guidance is available to develop precise, individualized interventions to improve sleep for optimal treatment of mental health disorders.

The first year of medical training (internship) is a rare circumstance marked by an abrupt increase in workload and shifting schedules that span the 24-h day. Additionally, the prevalence of depression increases sharply after the start of intern year^18,19^. Therefore, internship can act as a prospective model to more fully understand the relationship between sleep variability and mood for a broader population.

To precisely capture the variability of sleep over a longitudinal time course, methods that are both passive and objective are required. Actigraphy uses a wrist-worn accelerometer to collect motion data, and validated algorithms are applied to this data to distinguish sleep from wake^20,21^. Unfortunately, although actigraphy is an effective, well-verified method to evaluate sleep over days to weeks, some limitations can interfere with use in a large population under extremely demanding work schedules. Traditional actigraphs are expensive and typically lack wireless data transmission capability, which can limit both the duration of recording and size of the study population.

Technological advances in wrist-worn sensors provide the opportunity to objectively measure sleep through passive recording, in real time, with minimal expense or user burden^22,23^. Therefore, wrist-worn, multisensory consumer sleep tracking devices can now provide estimates of sleep patterns over extensive time durations in individuals under demanding circumstances such as medical training. Additionally, mobile platforms allow for real-time input of self-report symptoms^22^. Therefore, use of current technology provides the opportunity to more comprehensively characterize sleep, while simultaneously assessing mood, to identify the specific sleep disturbances that contribute to depression. Already, a small study using wearable and mobile technology (*N* = 33) demonstrated that short sleep duration and advances in sleep wake schedule in excess of 3 h (compared to sleep before intern year) were significant predictors of next day mood^24^.

Therefore, utilizing a sample of over 2000 subjects and a multisensory consumer sleep tracking device, the goals of the present study were to: 1) characterize the changes in objective, longitudinally monitored sleep with the transition into internship, 2) identify the specific objective sleep characteristics, including variability, associated with depression over the course of the intern year, and 3) evaluate the impact of day-to-day changes in objective sleep duration and sleep-wake timing on mood the next day. We hypothesized that decreased sleep duration and increased variability in sleep-wake timing would accompany the transition into internship and that shorter sleep duration and greater variability in sleep duration and timing would be associated with lower mood and more depressive symptoms.

## Results

### Sleep measures before and during internship

**Fig. 1:**
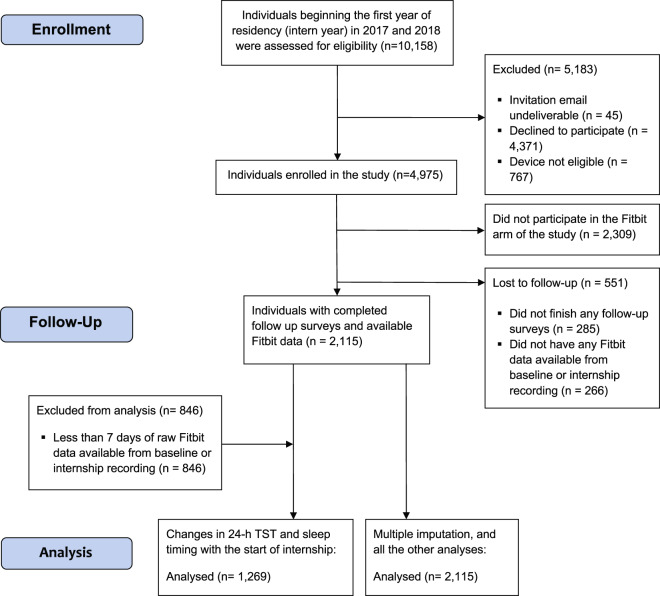
Study flow diagram. Flow diagram detailing subject inclusion from enrollment through follow-up and analysis.

The study cohort was comprised of 2115 (56% female; age 27.5 ± 2.4 years) interns (see Fig. 1 for details of subject inclusion). The mean of their baseline PHQ-9 scores and average internship PHQ-9 scores were 2.59 (±2.85) and 6.09 (±3.91) (Table 1). An average of 17 (±12) and 115 (±111) days of sleep recording were collected during the baseline period and intern year, respectively.

**Table 1:** Cohort demographics (*N* = 2115).

Age (years), mean (SD)	27.46 (2.43)	Specialty, *N* (%)	
Sex (female), *N* (%)	1196 (56.55)	Internal medicine	450 (21.28)
Baseline PHQ-9, mean (SD)	2.59 (2.85)	Surgery	186 (8.79)
Average internship PHQ-9, mean (SD)	6.09 (3.91)	Ob/Gyn	140 (6.62)
Baseline days of sleep recorded, mean (SD)	16.97 (11.80)	Pediatrics	287 (13.57)
Internship days of sleep recorded, mean (SD)	114.74 (110.51)	Emergency medicine	209 (9.88)
Race, *N* (%)		Med/Peds	52 (2.46)
White	1256 (59.39)	Family practice	185 (8.75)
Black/African American	97 (4.59)	Anesthesiology	110 (5.20)
Hispanic/Latino	74 (3.50)	Neurology	34 (1.61)
Asian	444 (20.99)	Otolaryngology	36 (1.70)
Arab/Middle Eastern	41 (1.94)	Transitional	69 (3.26)
Other/Multiracial/Not reported	203 (9.60)	Other/Not reported	240 (11.35)

*N* number of subjects, *SD* standard deviation.

With the onset of internship stress, training physicians experienced a significant reduction in average 24-h TST (17 min, *p* < 0.001, Cohen’s *d* = −0.42) and an advance in sleep timing, with median wake time moving nearly an hour earlier (*p* < 0.001, Cohen’s *d* = −0.76), and median bedtime moving forward around half an hour (*p* < 0.001, Cohen’s *d* = −0.47) after the start of internship (Table 2). Additionally, there was a significant increase in the standard deviation of sleep duration (16 min, *p* < 0.001, Cohen’s *d* = 0.63) and timing (bedtime, 1 h 53 min, *p* < 0.001, Cohen’s *d* = 1.47; wake time, 1 h 30 min, *p* < 0.001, Cohen’s *d* = 1.46) with the transition to intern year (Table 2). All comparisons passed the Bonferroni correction significance level (*p* = 0.05/6 = 0.008).

**Table 2:** Sleep parameters at baseline and during intern year (*N* = 1269*).

Sleep characteristic	Baseline	Internship	*t*	*p*	Cohen’s *d*
24-h TST, mean	6 h 43 min	6 h 26 min	−16.26	<0.001	−0.42
Bedtime, median	11:43 pm	11:10 pm	−16.19	<0.001	−0.47
Wake time, median	7:25 am	6:28 am	−21.95	<0.001	−0.76
24-h TST, SD	1 h 15 min	1 h 31 min	20.48	<0.001	0.63
Bedtime, SD	1 h 39 min	3 h 32 min	41.35	<0.001	1.47
Wake time, SD	1 h 44 min	3 h 14 min	42.59	<0.001	1.46

*Baseline (prior to internship) and internship sleep characteristics of individuals with at least 7 days of Fitbit data at both time points.

*N* number of subjects, *t* *t* statistic for within-subjects *t*-test, *p*-value statistical significance, Cohen’s *d*, effect size, SD, standard deviation, *TST* total sleep time, *h* hours, *min* minutes.

### Sleep predictors of depression

Multivariable linear regression models adjusted for age and sex were constructed to determine which sleep characteristics were associated with mean PHQ-9 depressive symptom score during intern year. Independent models examined each sleep parameter and its standard deviation separately, and all sleep parameters were considered simultaneously in the full model.

The mean PHQ-9 scores during intern year among the subjects ranged from 0 to 25.5. After inverse normalizing transformation, the scores ranged from −3.5 to 3.5. On average, for every 1 h decrease in 24 h TST, PHQ-9 score worsened by 0.11 points (transformed value, same below; *p* < 0.001). An even larger effect size was observed for variability in sleep duration; while controlling for 24-h TST, for every 1-h increase in the standard deviation of 24-h TST, PHQ-9 worsened by 0.4 points on average (*p* = 0.001). Median bedtime (*b* = 0.068, *p* = 0.015) but not median wake time (*b* = −0.012, *p* = 0.64) was associated with depression, with later bedtimes associated with higher depressive symptom scores, i.e., more depressive symptoms. In contrast, larger variability in wake time (*b* = 0.081, *p* = 0.005), but not bedtime (*b* = 0.037, *p* = 0.13), was associated with higher depressive symptom scores. After Bonferroni correction (significance level = 0.05/6 = 0.008), 24-h TST, the standard deviation of 24-h TST, and the standard deviation of wake time remained significantly associated with PHQ-9 score. See details in Table 3.

**Table 3:** Sleep predictors of PHQ-9 (*N* = 2115*).

Independent models	beta	*p*	Adjusted *R*^2^	beta	*p*	Adjusted *R*^2^	beta	*p*	Adjusted *R*^2^
24-h TST mean	−0.11	<0.001	0.010				−0.15	<0.001	0.014
24-h TST SD				0.18	0.11	0.00574	0.4	0.0012	
Bedtime median	0.068	0.015	0.0072				0.056	0.053	0.0079
Bedtime SD				0.05	0.034	0.0066	0.037	0.13	
Wake time median	−0.012	0.64	0.0046				−0.025	0.32	0.0079
Wake time SD				0.075	0.0076	0.0079	0.081	0.0049	
Full models	beta	*p*	Adjusted *R*^2^	beta	*p*	Adjusted *R*^2^	beta	*p*	Adjusted *R*^2^
24-h TST mean	−0.13	0.09	0.010				−0.23	0.012	0.015
Bedtime median	0.0057	0.94					−0.081	0.32	
Wake time median	0.039	0.58					0.11	0.2	
24-h TST SD				0.069	0.56	0.0099	0.33	0.016	
Bedtime SD				−0.27	0.021		−0.25	0.049	
Wake time SD				0.39	0.0064		0.31	0.045	
Full models adjusted for step parameters	beta	*p*	Adjusted *R*^2^	beta	*p*	Adjusted *R*^2^	beta	*p*	Adjusted *R*^2^
24-h TST mean	−0.11	0.16	0.015				−0.20	0.029	0.020
Bedtime median	0.022	0.77					−0.055	0.51	
Wake time median	0.018	0.80					0.074	0.37	
Step mean, × 1000	−0.027	<0.001					−0.034	0.0049	
24-h TST SD				0.065	0.59	0.011	0.34	0.012	
Bedtime SD				−0.27	0.023		−0.22	0.085	
Wake time SD				0.39	0.0069		0.27	0.078	
Step SD				−0.0011	0.049		7.2 × 10^−4^	0.40	

*N* number of subjects, *beta* beta estimate, *p* statistical significance, adjusted *R*^2^ coefficient of determination, *TST* total sleep time, *SD* standard deviation.

*Imputed data of all subjects in the cohort. Multivariable linear regression determining the association between baseline and internship sleep characteristics and average PHQ-9. Models adjusted for age and sex.

When all sleep factors were taken into consideration together in the full model, lower mean 24-h TST and bedtime variability and greater variability in 24-h TST and wake time were associated with higher depressive symptom scores. Overall, the variability of the sleep measures (24-h TST SD, bedtime SD and wake time SD) and mean levels of sleep measures had similar predictive value for depressive symptom scores (both adjusted *R*^2^ = 0.010). Combining all six factors together increased the adjusted *R*^2^ to 0.015. When further adjusting for mean and SD of daily steps, the effect of sleep parameters did not change significantly (Table 3).

**Fig. 2:**
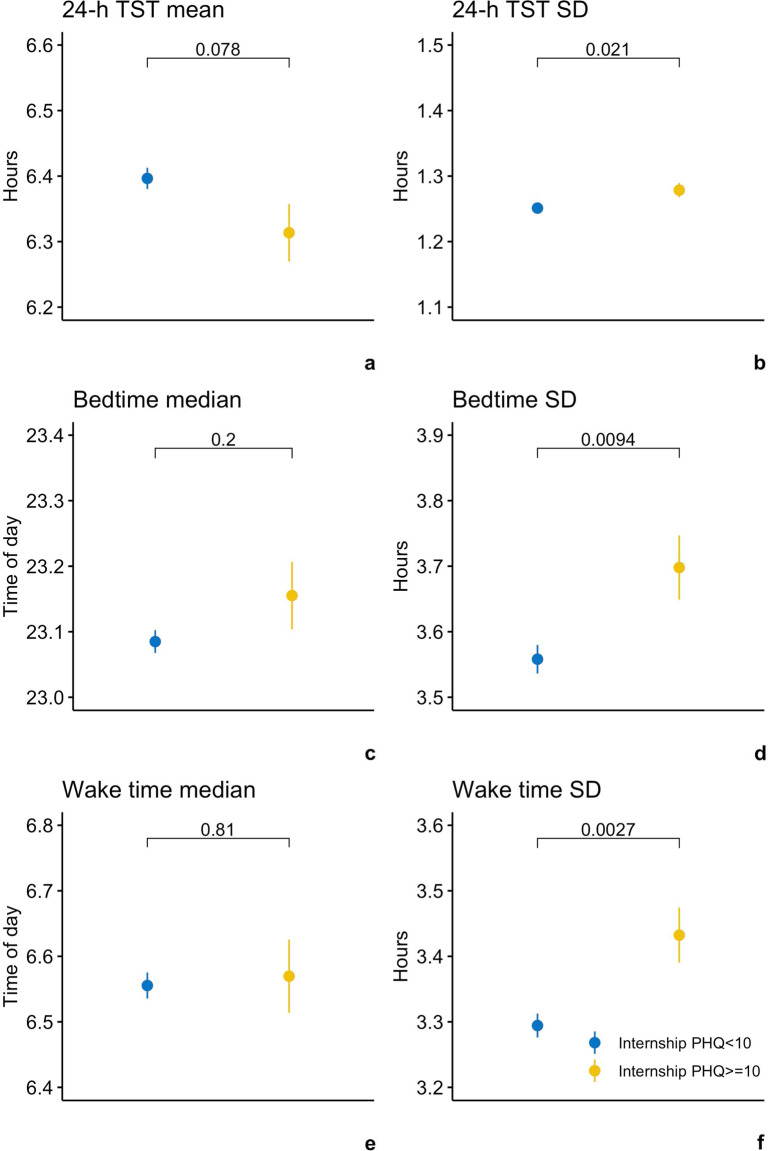
Sleep parameters comparison between non-depressed and depressed subjects during internship. Comparison of the sleep parameters (**a** 24-h TST mean; **b** 24-h TST SD; **c** Bedtime median; **d** Bedtime SD; **e** Wake time median; **f** Wake time SD) between non-depressed (*N* = 1757) and depressed (*N* = 358) subjects during internship, through two sample *t*-tests. TST total sleep time, SD standard deviation.

For a clearer data presentation, a secondary analysis of two sample *t*-tests were used to compare the objective sleep measures between depressed and non-depressed subjects. Out of 2115 subjects, 358 subjects had average internship PHQ-9 scores above the PHQ depression criteria (≥10). Compared to the remaining 1757 non-depressed subjects, they did not differ significantly in the mean or median of any sleep measures (24-h TST mean: 6.31 h vs. 6.40 h, *t* = −1.77, *p* = 0.078; bedtime median: 11:09 pm vs. 11:05 pm, *t* = 1.29, *p* = 0.20; wake time median: 6:34 am vs. 6:33 am, *t* = 0.24, *p* = 0.81), but had significantly greater variability in all three measures (24-h TST SD: 1.29 h vs. 1.25 h, *t* = 2.32, *p* = 0.021; bedtime SD: 3.70 h vs. 3.56 h, *t* = 2.61, *p* = 0.0094; wake time SD: 3.43 h vs. 3.29 h, *t* = 3.02, *p* = 0.0027) (Fig. 2). Wake time SD remained to be significantly different between depressed and non-depressed subjects after Bonferroni correction (significance level = 0.05/6 = 0.008).

### Daily effects of sleep measures on mood

Next, to better understand the temporal role of sleep timing and duration on mood the following day, a linear mixed model was used. The model was adjusted for age, sex, steps, and pre-internship factors as seen in Table 4.

**Table 4:** Day-to-day sleep predictors of next day mood.

	beta	95% CI	*p*
Intercept	0.69	0.11 to 1.28	0.020
*Between-subject effects (N = 2115)*			
Age	0.0061	−0.0080 to 0.020	0.40
Sex	−0.079	−0.15 to −0.0086	0.028
Mood, baseline	0.67	0.63 to 0.70	<0.001
24-h TST, baseline	−0.07	−0.13 to −0.013	0.015
Bedtime, baseline	−0.037	−0.089 to 0.015	0.16
Wake time, baseline	0.019	−0.030 to 0.069	0.45
Steps × 1000, baseline	−0.0045	−0.016 to 0.0067	0.43
*Within-subject effects (observations = 769,860)*			
Mood, previous day	0.081	0.079–0.084	<0.001
Steps × 1000, same day	1.5 × 10^−4^	4.7 × 10^−5^ to 2.5 × 10^−4^	0.004
Δ Steps	−1.9 × 10^−5^	−0.90 × 10^−5^ to 5.1 × 10^−5^	0.59
24-h TST, previous day	0.056	0.048 to 0.063	<0.001
Bedtime, previous day	−0.069	−0.076 to −0.062	<0.001
Wake time, previous day	0.089	0.082 to 0.096	<0.001
Δ 24-h TST	−0.011	−0.013 to −0.0083	<0.001
Δ Bedtime	0.0012	−5 × 10^−4^ to 0.0029	0.16
Δ Wake time	−0.0043	−0.0062 to −0.0024	<0.001
*Model fit statistics*			
ICC	0.30		
AIC	2,499,351.992		

Linear mixed modeling assessment of day-to-day changes in sleep characteristics on mood. Designating the day of mood assessment as d, sleep one (d-1) and two (d-2) nights prior to the mood measurement were considered. Δ, the absolute value of the difference of each sleep parameter on nights d-1 and d-2; beta, beta estimate.

*CI* confidence interval, *p* statistical significance, *N* number of subjects, *TST* total sleep time, *ICC* intraclass correlation coefficient, *AIC* Akaike information criterion.

Increased previous day 24-h TST (*b* = 0.056, *p* < 0.001) and later wake time (*b* = 0.089, *p* < 0.001) were associated with improved next day mood. Conversely, later bedtime was associated with worse next day mood (*b* = −0.069, *p* < 0.001).

Additionally, variability in 24-h TST (*b* = −0.011, *p* < 0.001) and wake time (*b* = −0.0043, *p* < 0.001) were associated with decreased next day mood. Variability in bedtime from night to night did not display a statistically significant impact on mood (*p* = 0.16).

## Discussion

Through collection of objective sleep data over an extended time period, our work revealed that in medical trainees, reduced total sleep time and later bedtime, and even more prominently, greater variability in total sleep time and wake time, were associated with increased depression. On a daily basis, reduced sleep duration, later bedtime, earlier wake time, and larger shifts in total sleep time and wake time were detrimental to next day mood. These findings augment the current understanding of the relationship between sleep and mental health given the large scope of our project (more than 2000 participants), assessment of objective sleep measures for more than 100 days of recording through one entire year, and conceptualization of sleep parameters as both averages and measures of variability.

Intraindividual variability (IIV) quantifies the daily variation around the mean for sleep parameters measured over multiple days^3^ and greater IIV in sleep metrics may exert a negative impact on a variety of outcomes^3–6^. However, previous investigations that assess the relationship between IIV of sleep and depression are often limited by the use of self-report sleep measures^3,9^. When objective sleep tracking has been utilized, the duration of longitudinal recording was typically less than 1–2 weeks or the sample size was much smaller than our cohort^10–12,14–17^. Therefore, the extreme work circumstances imposed on interns provide a model to comprehensively evaluate the impact of sleep variability on mood, which might be difficult to capture with research in naturalistic conditions among the general population.

Additionally, we used momentary assessment methodology to measure mood on a daily basis. Mood has been previously shown to vary day-to-day in the 72-h following overnight call^25^; therefore, usual measures that are vulnerable to recall bias are unlikely to appropriately characterize mood disturbances in this group. Furthermore, daily mood evaluation allowed us to replicate and extend on our prior work that assessed within subject effects of sleep on next day mood in a much smaller cohort of interns^24^ as well as similar work in other populations^26–29^.

As hypothesized, objectively measured shorter sleep duration was associated with increased depression scores (PHQ-9) during intern year. This extends previous findings by our group and others that demonstrated that short sleep duration is associated with elevated depression scores in medical trainees^18,30,31^. However, variability in sleep duration demonstrated an even stronger influence on PHQ-9 score, with a robust relationship between the standard deviation of sleep duration and depression scores, despite adjustment for 24-h TST. A similar finding was observed in a non-intern population that assessed sleep diary data and demonstrated a more than 2-fold increase in the odds of depression with every hour increase in the standard deviation of TST^9^.

With respect to sleep timing, bedtime but not wake-up time was associated with depression, with later bedtime associated with increased PHQ-9 scores. This finding may indicate that insomnia of sleep onset or evening chronotype is associated with worse mood during internship, given the known association between delayed sleep-wake phase disorder and depression^32–34^. However, after adjusting for sleep duration, this association was no longer significant and suggests that sleep loss is a potential factor underlying this finding.

Greater variability in wake-up time was associated with worse depression scores while conversely, increased variability in bedtime improved depression scores. These findings should be considered in the context of the a priori knowledge that bedtime is more contingent on individual selection or biological propensity, while wake time is fixed by external demands^35^ and specific to our population, variable based on workload. In general populations, this concept is highlighted by social jet lag, which describes the pattern of later timing and lengthier duration of sleep on free days than on work or school days, and is most pronounced in individuals with an evening circadian preference^35^. Notably, our prior work in a smaller population also demonstrated a 1.5-h advance in wake time after start of intern year without compensatory earlier bedtimes^24^.

Therefore, one hypothesis to explain the association of improved depression scores with more variable bedtimes, is that in individuals who do not successfully modify their bedtime, greater variations in wake time result in more variable (and reduced) sleep durations, which is detrimental to mood. Conversely, individuals who successfully vary bedtime in response to changing wake times maintain more stable, and increased, sleep durations and therefore have improved mood. In support of this hypotheses, a previous study demonstrated that the Morningness-Eveningness Questionnaire score was positively associated with sleep duration in medical residents, such that earlier chronotypes slept longer durations^36^.

Importantly, we operationalized sleep variability in two ways. Firstly, as the standard deviations of 24-h TST, bedtimes and wake times noted above. Although used extensively to measure sleep variability in the current literature^3,37^, standard deviation quantifies overall variability as averaged over days. To capture variability on a more granular level reflective of the day-to-day changes that are most disruptive to the circadian timekeeping system, other methods are required such as the sleep regularity index (SRI)^37^. The SRI is the probability of the same state (wakefulness or sleep) at time points 24 h apart^38^. Although the SRI was not used here, in addition to standard deviation, we quantified variability as the absolute value of the difference of each sleep measure between consecutive days. Therefore, both overall variability and variability on a day-to-day basis were evaluated.

Next day mood was worsened by shorter sleep duration, earlier wake times, and later bedtimes, which extends on our previous findings in a much smaller sample of interns^24^. When controlling for prior day sleep duration, sleep timing and mood, day-to-day shifts in total sleep time and wake time were also associated with a reduction in next day mood; corroborating the coarser relationship observed between sleep variability and depression scores averaged across the study. Shifts in bedtime were not associated with an impact on next day mood and therefore, suggests that shifts in bedtime are relevant for mood only in the context of their effect on sleep duration.

Our findings support the conclusion that variability of various sleep measures within an individual (IIV) may be more detrimental to mental health (and other conditions) than insufficient sleep alone, potentially through circadian disruption^3^. Alertness and sleep are optimal in quality and duration when wakefulness is attempted during the time of high circadian alerting signal and sleep coincides with the period of pineal melatonin secretion and reduced core body temperature. When external forces dictate behavioral rhythms out of alignment with our endogenous circadian rhythm, sleep, and mood deteriorate. The detriment of circadian disturbances to mood is evident in shift workers^39,40^, who undergo the most profound and chronic manifestation of circadian misalignment, but has also been associated with more indolent disruption, such as reduced amplitude of the circadian rest-activity rhythm^7^.

There are several limitations to this study. First, although the consumer sleep tracker used here, the Fitbit Charge 2™, has been validated against gold-standard polysomnogram and demonstrated performance that is similar to previously cited for research grade actigraphy, validation studies utilize single, overnight recordings that include only the main sleep episode. Therefore, the translation of this performance to daytime sleep episodes in shift workers and shorter bouts of polyphasic sleep, requires further validation^41^. Additionally, most currently available consumer sleep trackers (including the model used for this study) automatically identify the time in bed window without user input of bedtime and wake time, a capability that also requires further verification in a shift work population. Despite possible limitations, which are shared by many research grade actigraphs, objective sleep estimation over an extended time period in individuals under extensive work strain would not have been feasible without capitalizing on the availability of an acceptable, unobtrusive device that passively records sleep. Second, we operationalized sleep duration as 24-h TST, which includes all the sleep episodes in the 24-h period; therefore, the contribution of polyphasic sleep patterns to mood and depression were not assessed in this study and are worthy of investigation. Third, while the temporal relationship between sleep variability and depression can be valuable for applications including early detection and prediction, the potential of unmeasured factors, such as timing of physical activity and caffeine consumption, confounding the relationship preclude drawing conclusions about causality. We are hopeful that future randomized controlled trials will definitively assess whether decreasing sleep variability reduces depression. Fourth, while significant, the overall proportion of the depression score variance explained by sleep variability was small. As depression is a highly multidetermined phenotype^42,43^ with early life experience, stressful life events, psychological and genomic factors all playing important roles. In the context of these factors, one goal of the study was to compare the influence of sleep variability to mean levels of sleep on depression.

Recent changes have allowed for flexibility in the previously mandated standard duty hours that were implemented by the Accreditation Council for Graduate Medical Education (ACGME) in July 2011. The potential impact of relaxing duty hour restrictions was assessed in the Individualized Comparative Effectiveness of Models Optimizing Patient Safety and Resident Education (iCOMPARE) trial, which recently demonstrated that chronic sleep loss and sleepiness were similar among interns in flexible programs and standard programs^44^. Further, no detriment to patient safety outcomes was observed^45^. However, by leveraging current technological advances of multisensory consumer sleep trackers and digital, momentary mood assessments, we were able to detail more granular relationships between sleep, depression, and daily mood which reveals the relevance of sleep regularity for optimal mental health in interns.

Our findings provide a necessary foundation to inform institutional scheduling structures and guide self-management measures to improve sleep and circadian alignment within the confines of a demanding workload with the ultimate goal of optimizing mental health.

Additionally, these findings have implications far beyond medical trainees, as a growing body of work has started to evaluate the contribution of day-to-day sleep variability to depression and other various aspects of health^3–6,9^. Therefore, the results presented here are an extension of the ample work evaluating the relationships between sleep and mood and provide significant insight into longitudinal sleep patterns and depression.

Our current society is connected on a global scale, which offers opportunities for work and social networking across the 24-h day, oftentimes at the expense of sufficient, consistent sleep. Therefore, even in the context of small effect sizes, our findings have clinical value. By identifying variability in sleep duration and timing as a potential factor associated with mood, this modifiable behavior could be considered more broadly as part of a multifaceted approach to optimize mental health in general adult populations.

## Methods

### Study design and participants

The Intern Health Study is a multisite prospective cohort study that follows training physicians through internship (for details, see Guille^46^ and Sen^19^).

Two to three months prior to the start of the of residency, 4975 subjects across 430 institutions starting residency in 2017 and 2018 were invited to participate in the Intern Health Study. A cohort of 2115 subjects with survey, daily mood, and Fitbit data was used for the current analysis.

Prior to the start of internship, subjects completed a baseline survey and subsequently completed assessments every 3 months during the intern year through a mobile app. A multisensory (motion and heart rate) consumer sleep tracking device (The Fitbit Charge 2™) was worn on the wrist to measure sleep continuously before and during intern year. Additionally, through our mobile app, mood valence was assessed daily through a push notification sent to interns at a user-specified time between 5 pm to 10 pm daily with a scale from 1 to 10 (developed by Remedy Health Media LLC, New York, NY, Foreman 2011)^47^. See Supplementary Fig. 1 for the detailed protocol and Supplementary Fig. 2 for mood assessment interface. This study was approved by the University of Michigan IRB and all subjects provided informed consent after receiving complete description of the study.

### Assessments

Baseline and quarterly surveys allowed for extraction of demographics and other measures, as well as depressive symptoms with the patient health questionnaire (PHQ-9). The 9-item patient health questionnaire (PHQ-9) is a self-report component of the primary care evaluation of mental disorders inventory. The diagnostic validity of the PHQ-9 has been demonstrated as comparable to clinician-administered assessments^48,49^. For each of the nine depressive symptoms included in diagnostic and statistical manual of mental disorders (DSM-5)^50^, subjects were asked whether, during the previous 2 weeks, the symptom had bothered them “not at all”, “several days”, “more than half the days”, or “nearly every day”. Each item yields a score of 0–3, making the total score ranges from 0 to 27. PHQ depression, defined by a score of 10 or greater on the PHQ-9, has moderate sensitivity (88%) and specificity (85%) for a diagnosis of major depression disorder^51^.

Internship PHQ-9 score was calculated by averaging PHQ-9 scores across all available quarterly assessments. Daily mood was quantified by the response to following query: “On a scale of 1 (lowest) to 10 (highest), how was your mood today?”

The Fitbit Charge 2™contains an accelerometer and photoplethysmography sensor and applies proprietary algorithms to motion and heart rate features to quantify sleep. Though not an FDA cleared medical device, the Fitbit Charge 2™ has been compared to in laboratory polysomnogram (PSG) and demonstrates 0.96 sensitivity (accuracy to detect sleep) and 0.61 specificity (accuracy to detect wake) in healthy adults^52^. Summary sleep metrics demonstrate that the Fitbit Charge Fitbit Charge 2™ overestimated PSG total sleep time (TST) by 9 ± 24 min and underestimated PSG sleep onset latency (SOL) by 4 ± 9 min, but was similar to PSG in the determination of wake after sleep onset (WASO)^52^.

Consistent with prior studies, sleep episodes were assigned to a day when the wake time occurred on that day. For days with two or more sleep periods, the longest bout was designated as the main sleep episode. In estimating sleep duration, the TST for all sleep episodes in one day was summed to capture both the main sleep episode and naps (24-h TST).

In addition to 24-h TST, daily main sleep episode bedtime and wake time were also extracted for each day of Fitbit use. The mean/median and standard deviation (SD) of each sleep measure during the internship year comprised the objective sleep characteristics of interest for analysis.

In parallel, accelerometry-based daily step counts, treated as a proxy for physical activity, were recorded from Fitbit use.

### Statistical methods

All statistical analyses were conducted with the use of R (The R Foundation, Vienna, AUT)^53^.

To assess changes in average TST and its variability, as well as the median and variability of sleep timing with the start of internship stress, we utilized within-subjects paired *t*-tests. We tested for changes in 24-h TST and timing of the main sleep episode between baseline (prior to internship) and intern year on subjects with at least 7 days of raw Fitbit data for both time points (*N* = 1269).

On the full set of subjects (*N* = 2115), multiple imputation using predictive mean matching was applied to impute the missing baseline demographics, and then daily mood and Fitbit measures during internship, with the R package mice^54^.

To determine the relationship between objective sleep measures and depressive symptoms during internship, we employed multivariable linear regression models adjusted for age and sex, with the mean level or the standard deviation of each Fitbit sleep measure during internship as predictors of average internship PHQ-9 score. We also assessed three full models which consider all the mean level of sleep parameters, all the standard deviation of sleep parameters and all the six parameters simultaneously. To address the potential confounding effect of physical activity, we again assessed these full models with additional covariates including the mean and standard deviation of daily step counts (in the unit of 1000), which served as a proxy of physical activity. As raw internship PHQ-9 score was left-skewed, and the residuals were not normally distributed, inverse normalizing transformation was applied to produce near-normal distributions. To provide a clearer presentation of the relationship, a secondary analysis of two sample *t*-tests were used to compare the objective sleep measures of the subjects whose average internship PHQ-9 met criteria for depression (PHQ score ≥ 10) with those of the non-depressed subjects.

The impact of day-to-day changes in sleep characteristics on next day mood (measured on a Likert scale of 1–10 as previously described), was evaluated with linear mixed modeling, allowing for the simultaneous assessment of between-subjects and within-subjects effects^55,56^. Designating the day of mood assessment as d, sleep one (d-1) and two (d-2) nights prior to the mood measurement were considered. To assess the effect of sleep measure variability on mood, we assessed the absolute value of the difference of each sleep measure (24-h TST, main sleep episode bedtime, and main sleep episode wake time) on nights d-1 and d-2, Δ = |*s*_(d-1)_ − *s*_(d-2)_|. Models were adjusted for age, sex, baseline and previous day mood, and 24-h TST^24^, baseline and previous night main sleep episode bedtime and wake time, baseline and same day steps (in the unit of 1000), and absolute change of steps (in the unit of 1000) from previous day.

To correct for multitesting, Bonferroni corrections were applied for the paired *t*-tests assessing the change in TST and timing with the start of internship stress, the independent models examining sleep predictors of depression, and the two-sample *t*-tests comparing depressed and non-depressed subjects.

### Reporting summary

Further information on research design is available in the Nature Research Reporting Summary linked to this article.

## Supplementary information

Supplementary Information

Reporting Summary

## Data Availability

The de-identified data from Intern Health Study that support the findings described here are available from the corresponding author upon reasonable request.

## Code availability

Code for data preprocessing and statistical analysis is available upon reasonable request.
