# Supplementary material for: Day-to-day variability in sleep parameters and depression risk: a prospective cohort study of training physicians
Source: NPJ Digit Med. 2021 Feb 18;4:28. doi: 10.1038/s41746-021-00400-z (PMC7892862; doi:10.1038/s41746-021-00400-z)
Supplement: Supplementary file 1 — Supplementary Information [file 41746_2021_400_MOESM1_ESM.pdf]

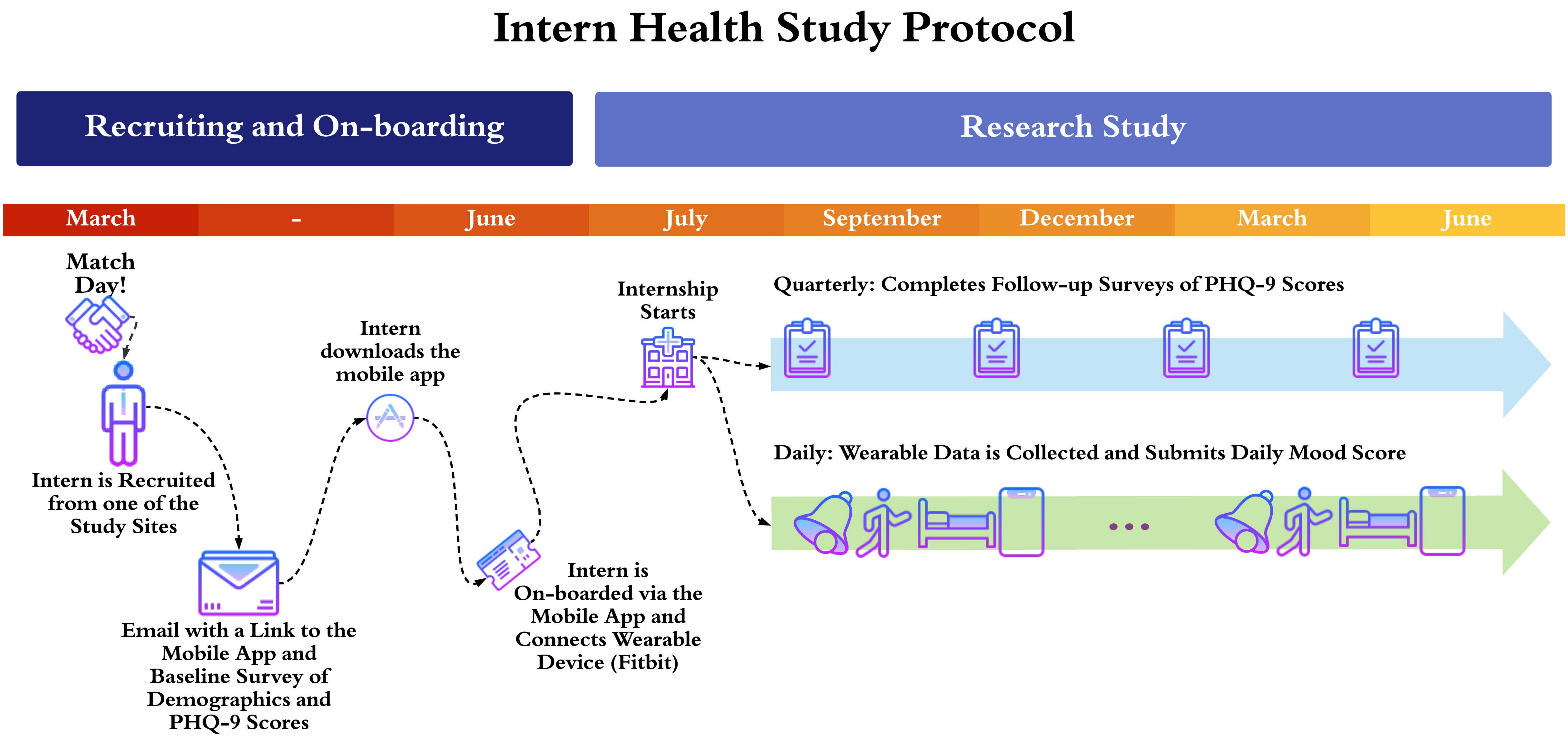

Supplementary Figure 1: Study Protocol of Intern Health Study

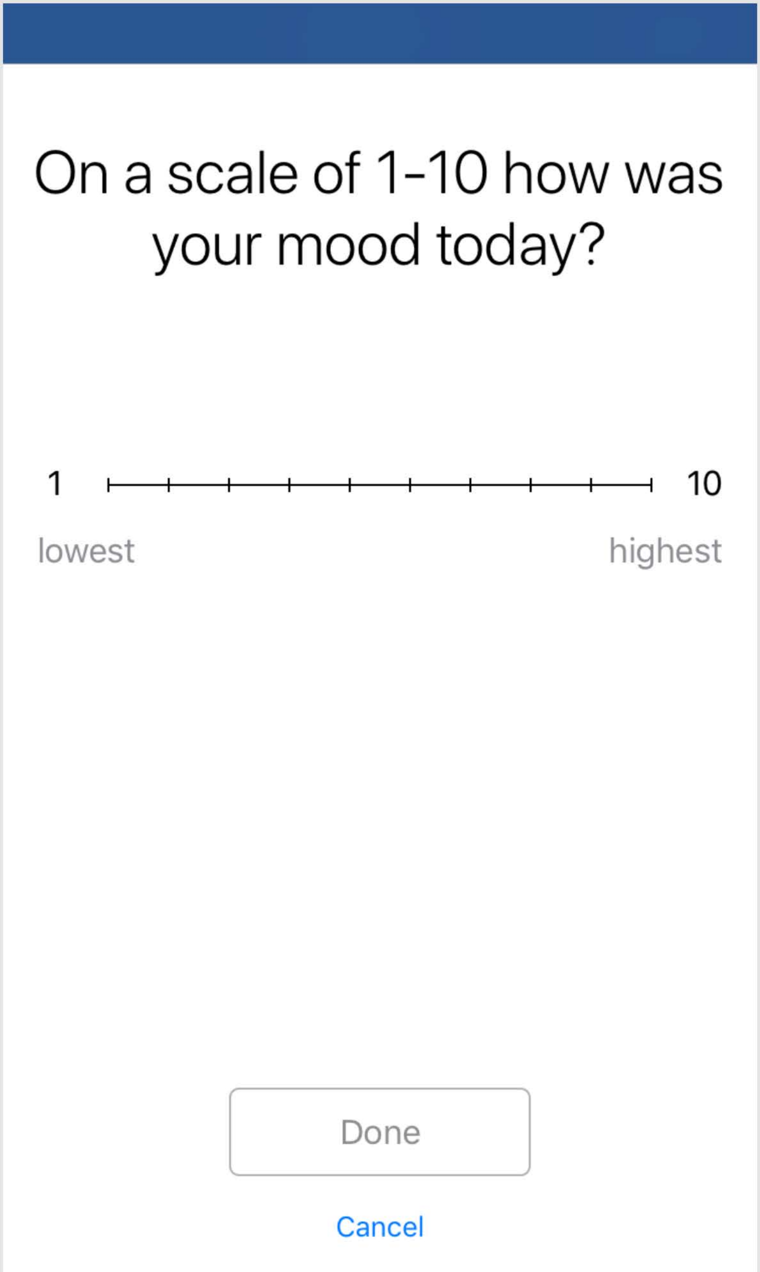

Supplementary Figure 2: Mood Assessment Interface
