# Supplementary material for: Day-to-day variability in sleep parameters and depression risk: a prospective cohort study of training physicians
Source: NPJ Digit Med. 2021 Feb 18;4:28. doi: 10.1038/s41746-021-00400-z (PMC7892862; doi:10.1038/s41746-021-00400-z)
Supplement: Supplementary file 2 — Reporting Summary [file 41746_2021_400_MOESM2_ESM.pdf]

nature research

Corresponding author(s): Yu Fang

Last updated by author(s): Jan 8, 2021

Reporting Summary

Nature Research wishes to improve the reproducibility of the work that we publish. This form provides structure for consistency and transparency in reporting. For further information on Nature Research policies, see our Editorial Policies and the Editorial Policy Checklist.

Statistics

For all statistical analyses, confirm that the following items are present in the figure legend, table legend, main text, or Methods section.

n/a	Confirmed	
☐	☒	The exact sample size (n) for each experimental group/condition, given as a discrete number and unit of measurement
☐	☒	A statement on whether measurements were taken from distinct samples or whether the same sample was measured repeatedly
☐	☒	The statistical test(s) used AND whether they are one- or two-sided<br>*Only common tests should be described solely by name; describe more complex techniques in the Methods section.*
☐	☒	A description of all covariates tested
☐	☒	A description of any assumptions or corrections, such as tests of normality and adjustment for multiple comparisons
☐	☒	A full description of the statistical parameters including central tendency (e.g. means) or other basic estimates (e.g. regression coefficient) AND variation (e.g. standard deviation) or associated estimates of uncertainty (e.g. confidence intervals)
☐	☒	For null hypothesis testing, the test statistic (e.g. F, t, r) with confidence intervals, effect sizes, degrees of freedom and P value noted<br>*Give P values as exact values whenever suitable.*
☒	☐	For Bayesian analysis, information on the choice of priors and Markov chain Monte Carlo settings
☒	☐	For hierarchical and complex designs, identification of the appropriate level for tests and full reporting of outcomes
☐	☒	Estimates of effect sizes (e.g. Cohen's d, Pearson's r), indicating how they were calculated

Our web collection on statistics for biologists contains articles on many of the points above.

Software and code

Policy information about availability of computer code

Data collection	Survey data was collected on line through Qualtrics embedded in the mobile App 'Intern+'. Wearable data was collected through the mobile App 'Intern+'.
Data analysis	All statistical analyses were conducted with the use of R (packages: base packages, lme4, lmerTest, effsize, ggplot2).

For manuscripts utilizing custom algorithms or software that are central to the research but not yet described in published literature, software must be made available to editors and reviewers. We strongly encourage code deposition in a community repository (e.g. GitHub). See the Nature Research guidelines for submitting code & software for further information.

Data

Policy information about availability of data

All manuscripts must include a data availability statement. This statement should provide the following information, where applicable:

- Accession codes, unique identifiers, or web links for publicly available datasets
- A list of figures that have associated raw data
- A description of any restrictions on data availability

The de-identified data from Intern Health Study that support the findings described here are available from the corresponding author upon reasonable request. Code for data preprocessing and statistical analysis is available upon reasonable request.

Field-specific reporting

Please select the one below that is the best fit for your research. If you are not sure, read the appropriate sections before making your selection.

☐ Life sciences ☒ Behavioural & social sciences ☐ Ecological, evolutionary & environmental sciences

For a reference copy of the document with all sections, see nature.com/documents/nr-reporting-summary-flat.pdf

Behavioural & social sciences study design

All studies must disclose on these points even when the disclosure is negative.

Study description	The Intern Health Study is a multi-site prospective quantitative cohort study that follows training physicians through internship year.
Research sample	4975 eligible incoming residents in the United States in cohort 2017 and 2018 were enrolled in the Intern Health Study. In total, a representative group of 2115 subjects (56% female; Age 27.5 ± 2.4 years) who provided survey, daily mood, and Fitbit data and were included in the current analysis.
Sampling strategy	Subjects are drawn from incoming intern classes at university and community residency programs in the US. Lists of names and email addresses of eligible incoming interns to invite are provided by residency program directors at individual institutions, or obtained from publically available medical school match lists.
Data collection	Survey data was collected on line through Qualtrics embedded in the mobile App 'Intern+'. Wearable data was collected through the mobile App 'Intern+'.
Timing	The subjects were invited to the study and finished the baseline survey two to three months prior to the start of the of residency (July 1 in 2017 or 2018), and were asked to finish the follow-up survey every 3 months during the intern year (the last one was delivered in June 2018 or 2019). Wearable data was collected upon the enrollment and the last day of wearable data collection is June 30, 2018 or 2019.
Data exclusions	Data exclusion criteria was pre-established that subjects who was not a part of the Fitbit arm of the study (n = 2,309), or did out finish any follow-up survey (n = 285) or without any wearable data available from baseline or internship (n = 266).
Non-participation	551 participants lost to follow-up in the study after enrollment. When provided, reasons included losing the Fitbit, preference to not wear the Fitbit to sleep, or discontinuing their internship program.
Randomization	Randomization is not relevant to our study as there's no group allocation.

Reporting for specific materials, systems and methods

We require information from authors about some types of materials, experimental systems and methods used in many studies. Here, indicate whether each material, system or method listed is relevant to your study. If you are not sure if a list item applies to your research, read the appropriate section before selecting a response.

Materials & experimental systems

n/a	Involved in the study	
☒	☐	Antibodies
☒	☐	Eukaryotic cell lines
☒	☐	Palaeontology and archaeology
☒	☐	Animals and other organisms
☐	☒	Human research participants
☒	☐	Clinical data
☒	☐	Dual use research of concern

Methods

n/a	Involved in the study	
☒	☐	ChIP-seq
☒	☐	Flow cytometry
☒	☐	MRI-based neuroimaging

Human research participants

Policy information about studies involving human research participants

Population characteristics	See above.
Recruitment	Following the residency match in March, the study team obtains a list of names and email addresses for incoming interns from over 60 approved hospitals and medical schools across the US. Potential subjects are contacted via email with a description of the study and an invitation to participate. Invitations are extended on a rolling basis prior to the start of internship (July 1). Delays in the availability of the Intern Health Study mobile app resulted in a subset of potential subjects being invited to a web survey and DNA arm only (no wearable data). Initial versions of the app were also only compatible

	with iOS devices, so any incoming intern without an iPhone as their primary mobile device would not have been eligible to enroll.
Ethics oversight	This study was approved by the University of Michigan IRB and all subjects provided informed consent after receiving complete description of the study.

Note that full information on the approval of the study protocol must also be provided in the manuscript.
